# Evaluation of Bone Quality for Optimal Anchor Insertion Point in Arthroscopic Anterior Talofibular Ligament Repair Using Computed Tomography Hounsfield Unit Analysis

**DOI:** 10.7759/cureus.88225

**Published:** 2025-07-18

**Authors:** Munekazu Kanemitsu, Tomoyuki Nakasa, Yasunari Ikuta, Dan Moriwaki, Satoru Sakurai, Saori Ishibashi, Taro Chujo, Nobuo Adachi

**Affiliations:** 1 Department of Orthopaedic Surgery, Matsuyama Red Cross Hospital, Matsuyama, JPN; 2 Department of Artificial Joints and Biomaterials, Graduate School of Biomedical and Health Sciences, Hiroshima University, Hiroshima, JPN; 3 Department of Orthopaedic Surgery, Graduate School of Biomedical and Health Sciences, Hiroshima University, Hiroshima, JPN

**Keywords:** anterior talofibular ligament, arthroscopic repair, bone quality, chronic lateral ankle instability, hounsfield unit value

## Abstract

Background: The all-inside arthroscopic Broström procedure using suture anchors has gained popularity and yielded favorable clinical outcomes for chronic lateral ankle instability (CLAI). However, recurrent instability after surgery remains a significant concern, and one of the possible causes may be the loosening of the repaired ligament due to anchor dislocation or loosening by poor bone quality. The anchor insertion position for the anterior talofibular ligament (ATFL) is often proximal to its anatomical attachment because it is not fully observed under arthroscopy, and non-anatomical points may have poor bone quality. This study aimed to evaluate bone quality at anatomical and non-anatomical anchor insertion points in ATFL repair.

Methods: Forty-four ankles of 43 patients (21 men and 23 women; mean age 35.4 years) who underwent arthroscopic ATFL repair for CLAI were included. Anatomic attachment sites were identified using the fibular obscure tubercle (FOT) as an indicator. Hounsfield unit (HU) values were measured at the anatomical and proximal non-anatomical points of ATFL attachment at the fibula in preoperative computed tomography images and compared between these points.

Results: The HU value at the anatomical point was significantly higher than that at the non-anatomical point in both coronal and sagittal images (P<0.05). No significant differences in HU values at anatomical and non-anatomical points were observed between sexes or in ATFL remnant quality. The HU value in the point proximal 0-2 mm from the anatomical attachment of the ATFL was significantly higher than in the point proximal 6-8 mm from the anatomical point. Weak negative correlations between HU values and age were observed at the point proximal 4-8 mm from the anatomical attachment of the ATFL.

Conclusion: Anchor insertion into the non-anatomical attachment at the fibular side has a potential risk of anchor loosening or dislocation due to poor bone quality. To prevent this, anchor insertion should be inserted within 4 mm proximal to the anatomical attachment of the ATFL.

## Introduction

Acute ankle sprains are among the most common musculoskeletal injuries, and up to 70% of individuals who sustain an acute ankle sprain may develop residual physical disabilities, including chronic lateral ankle instability (CLAI) [1]. Among several surgical procedures for CLAI, arthroscopic Broström procedures using suture anchors have been widely used recently, with good clinical outcomes [2-8]. In this technique, the anchor is inserted into the fibula, and the anchor is sometimes dislocated or loosened, which may lead to the recurrence of the instability after surgery. Although various factors, such as hindfoot alignment and remnant quality, have been reportedly associated with the recurrence of instability [9-11], recurrent instability caused by anchor dislocation or loosening should be considered. In rotator cuff repair of the shoulder, poor bone quality may be a risk factor for anchor loosening and re-tear, and the same risk is possible with anterior talofibular ligament (ATFL) repair using anchors [12-17]. The subchondral bone plate in the attachment of the ATFL at the fibula side is thinner than that at the talar side, and there is an abrupt decrease in bone density 4 mm below the tidemark at the fibula attachment, suggesting a greater frequency of avulsion fractures of the fibula attachment [18]. In contrast, the uncalcified and calcified fibrocartilage at the ligament attachment is thicker than the surrounding area, because tension applied to the ligaments is transmitted to the bone [2]. This suggests that the bone is weak except at the ATFL attachment at the fibula; moreover, anchor insertion outside of the anatomic ATFL attachment may result in dislocation or loosening of the inserted anchor due to poor bone quality. However, the anchor insertion position is often proximal to the ATFL attachment in arthroscopic repair because ATFL attachment at the fibula is not fully observed under arthroscopy [19]. Therefore, evaluating the bone quality of the possible anchor insertion area is important for achieving good postoperative outcomes. 

Hounsfield unit (HU) values on computed tomography (CT) are effective for quantitatively assessing bone conditions [20]. HU values are defined to be 0 for water and -1000 HU for air, and correlate with bone density at the site of measurement [21]. Examining the HU values of possible anchor insertion locations will reveal inappropriate locations for anchor insertion from the viewpoint of bone quality. We hypothesized that measuring HU values at the fibula might determine the optimal anchor insertion position in arthroscopic ATFL repair. This study aimed to examine the difference in bone quality between the anatomic and non-anatomic points for anchor insertion by measuring HU values on CT images and to determine the optimal anchor insertion position in arthroscopic ATFL repair.

## Materials and methods

Participants

Forty-four ankles of 43 patients who underwent arthroscopic ligament repair for CLAI between April 2019 and November 2023 were included in this study. They comprised 21 men and 23 women, with a mean age of 35.4±14.2 years (14-58 years). One patient involved both ankles. CLAI was diagnosed by orthopedic specialists based on the history of ankle sprain, ankle instability, stress plain radiographs, magnetic resonance imaging, and arthroscopic findings. All patients included in this study underwent preoperative CT within one month of surgery. Patients with systemic diseases such as rheumatoid arthritis, deformity of the fibula due to trauma, and subfibular ossicles were excluded. This study was approved by the local ethical committee of our institution, and informed consent was obtained from all individual participants included in this study.

CT evaluation

**Figure 1:**
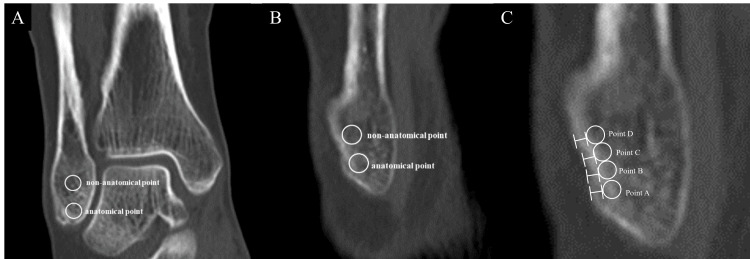
Measurement points. (A) HU values were measured at anatomical and non-anatomical points in coronal image. (B) HU values were measured at anatomical and non-anatomical points in sagittal image. (C) Points A to D were established from the fibular obscure tubercle (FOT). HU values and cortical bone thickness of each point were measured in sagittal image. HU: Hounsfield unit.

All patients underwent preoperative scans using a multidetector-row CT scanner (LightSpeed QX/I; GE Healthcare, Chicago, IL), which was calibrated monthly to ensure standardized image quality. Imaging parameters included a 512 × 512 matrix, 0-degree gantry tilt, 1.25-mm prospective slice thickness, 120 kV (peak) tube voltage, and 120-200 mA tube current. Two-dimensional images were reconstructed with a 25-cm field of volume, 1.25-mm retrospective slice thickness, and 0.63-mm overlap. The anatomical position was identified on the sagittal section image using the FOT as a reference. Then, the reference line of that slice was used to determine the measurement position on the coronal section image. In the coronal and sagittal planes, the anatomical attachment of the ATFL at the fibula was identified at 3.7 mm proximal from the fibular obscure tubercle (FOT) according to a previous report [22]. A non-anatomical point was defined as the 8-mm proximal point according to the previous report [23], and a 3 mm diameter region of interest (ROI) on a regular circle was established in the cancellous bone area at the anatomical and non-anatomical ATFL attachment. Then, HU values were measured using ShadeQuest/ViewRD-G V1.26 (Fujifilm Medical Co., Ltd., Tokyo, Japan) (Figures 1A, 1B). In addition, to further examine in detail, a ROI on a 2 mm diameter regular circle was established in the trabecular bone region at four locations proximal to the anatomical point (point A: 0-2 mm, B: 2-4 mm, C: 4-6 mm, and D: 6-8 mm), and the cortical bone thickness directly above the ROI at each point on the sagittal plane image was measured (Figure 1C). Because the insertion of the anchor in arthroscopic repair tends to be proximal, the ROI was set proximally to the anatomical position.

Arthroscopic evaluation

Based on the arthroscopic findings, ATFL remnants were classified into three categories: excellent, moderate, and poor according to a previous report [24]. For each remnant quality, the HU values at the anatomical and non-anatomical points were compared.

Statistical analysis

HU values of the two groups were compared using the Mann-Whitney U test. The multiple comparisons were performed using the Kruskal-Wallis test, and the Steel-Dwass test was used for a post-hoc test. Correlations between the HU ratios and several parameters were tested using Spearman's rank correlation coefficient. Statistical significance was set at p<0.05. r is defined as follows: r<0.3: no correlation, 0.3≦r<0.5: very weak correlation, 0.5 ≦r<0.7: correlation, 0.7 ≦r<0.9: strong correlation, and r≧0.9: very strong correlation.

## Results

**Figure 2:**
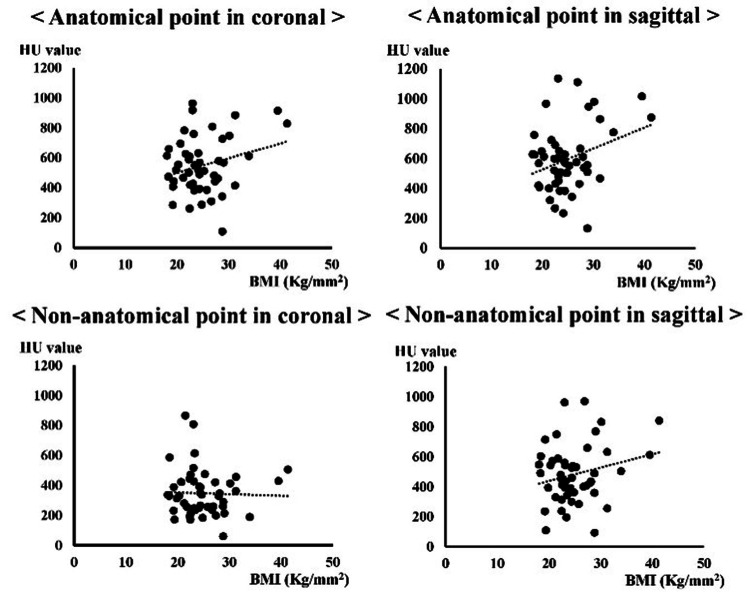
Correlations between the mean body mass index (BMI) and HU value. HU: Hounsfield unit. There was no significant correlation (anatomical point: coronal r=-0.03; p=0.83 and sagittal r=-0.10; p=0.51, non-anatomical point: coronal r=-0.12; p=0.43, sagittal r=-0.28; p=0.06). Spearman rank correlation coefficient was used. p-value<0.05 is considered significant. r<0.3: no correlation, 0.3≦r<0.5: very weak correlation, 0.5 ≦r<0.7: correlation, 0.7≦r<0.9: strong correlation, and r≧0.9: very strong correlation.

**Figure 3:**
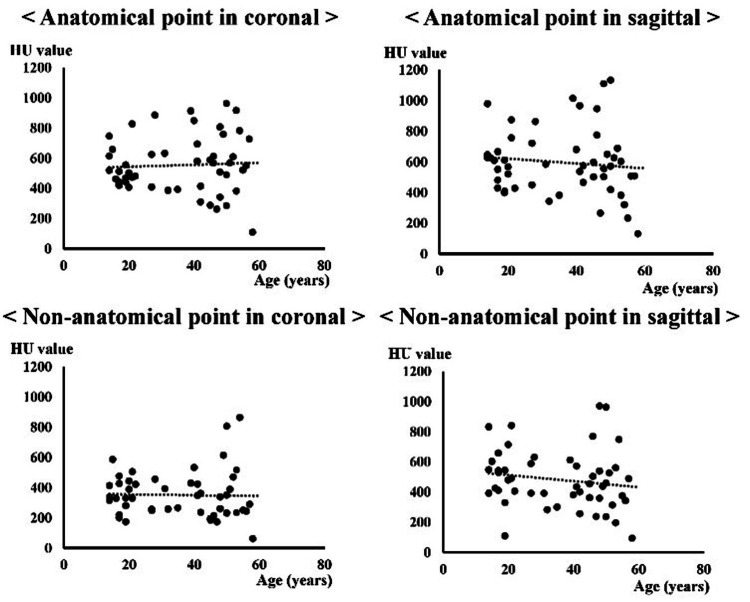
Correlations between age and HU value. HU: Hounsfield unit. There was no significant correlation (anatomical point: coronal r=0.14; p=0.38 and sagittal r=0.12; p=0.46, non-anatomical point: coronal r=-0.01; p=0.95 and sagittal r=0.13; p=0.42). Spearman rank correlation coefficient was used. p-value<0.05 is considered significant. r<0.3: no correlation, 0.3≦r<0.5: very weak correlation, 0.5 ≦r<0.7: correlation, 0.7 ≦r<0.9: strong correlation, and r≧0.9: very strong correlation.

In both the coronal and sagittal images, the mean HU values were significantly higher at the anatomical points than at the non-anatomical points (Table 1). The HU values of the anatomical or non-anatomical points on the coronal and sagittal images did not significantly differ between sexes (Table 2). Based on arthroscopic findings, the remnant quality was eight ankles, 20 ankles, and 16 ankles in the excellent, moderate, and poor categories, respectively. No significant differences in HU values were observed at the anatomical and non-anatomical points in each remnant (Table 3). Significant correlations between BMI and HU value were not observed (anatomical point: coronal: r=-0.03, p=0.83; sagittal: r=-0.10, p=0.51, non-anatomical point: coronal: r=-0.12, p=0.43; sagittal: r=-0.28, p=0.06) (Figure 2). The mean body mass index (BMI) was 24.6±5.1 kg/m^2^ (range: 18.4-39.5), and there were no significant correlations between age and HU value were not also observed (anatomical point: coronal: r=0.14, p=0.38; sagittal: r=0.12, p=0.46, non-anatomical point: coronal: r=-0.01, p=0.95; sagittal r=0.13, p=0.42) (Figure 3).

**Table 1:** Hounsfield unit (HU) values of the anatomical and the non-anatomical points in coronal and sagittal images. HU values are significantly higher at the anatomical points. Mann-Whitney U test was used. p-value<0.05 is considered significant.

	Anatomical point, mean±SD (range)	Non-anatomical point, mean±SD (range)	p-value
Coronal	566.4±184.0 (285.4-962.7)	356.5±132.2 (172.9-807.3)	<0.01
Sagittal	623.6±203.5 (325.0-1135.2)	488.3±184.9 (196.0-969.7)	<0.01

**Table 2:** Comparison of HU values between males and females. There is no significant difference. Mann-Whitney U test was used. p-value<0.05 is considered significant. HU: Hounsfield unit.

	Male, mean±SD (range)	Female, mean±SD (range)	p-value
Coronal			
Anatomical point	662.2±212.7 (262.3-962.7)	515.4±139.2 (288.1-884.6)	0.13
Non-anatomical point	391.6±158.5 (172.9-807.3)	324.6±95.4 (195.0-456.3)	0.12
Sagittal			
Anatomical point	655.7±248.5 (266.7-1135.2)	594.3±151.4 (325.0-967.3)	0.53
Non-anatomical point	521.5±235.8 (196.0-969.7)	458.0±119.3 (194.6-631.7)	0.64

**Table 3:** Comparison of HU values in the remnant quality of the anterior talofibular ligament. There is no significant difference. Mann-Whitney U test was used. p-value<0.05 is considered significant. HU: Hounsfield unit.

	Excellent, mean±SD (range)	Moderate, mean±SD (range)	Poor, mean±SD (range)	p-value
Coronal				
Anatomical point	532.2±165.3 (407.0-807.9)	543.3±207.4 (262.3-962.7)	612.3±162.0 (414.5-913.1)	0.39
Non-anatomical point	328.8±132.1 (218.0-587.0)	357.0±158.6 (188.7-807.3)	369.9±97.5 (280.7-614.7)	0.55
Sagittal				
Anatomical point	613.8±224.4 (420.0-1111.5)	607.2±231.6 (343.8-1135.2)	649.9±161.7 (399.9-1015.8)	0.60
Non-anatomical point	543.3±230.6 (236.8-969.7)	449.6±207.4 (196.0-962.8)	509.2±162.0 (256.2-840.4)	0.28

Regarding HU values at the points between anatomical and non-anatomical points, the HU value in point A was significantly higher than in point D. However, there was no significant difference in cortical bone thickness between each point (Tables 4, 5). There were no significant differences between males and females at each point (Table 6). Weak negative correlations between HU values and age were observed at point C (r=-0.32, p=0.03) and point D (r=-0.30, p=0.048), but no correlations were observed at point A (r=-0.17, p=0.26) and point B (r=-0.26, p=0.09).

**Table 4:** Comparison of HU values and cortical bone thickness. A significant difference was observed in HU values. Kruskal-Wallis test was used. p-value<0.05 is considered significant. HU: Hounsfield unit.

	Point A, mean±SD (range)	Point B, mean±SD (range)	Point C, mean±SD (range)	Point D, mean±SD (range)	p-value
HU value	775.8±218.7 (242.0-1164.1)	739.4±220.6 (297.3-1177.4)	677.3±194.5 (373.1-1164.9)	681.7±187.0 (394.4-1137.1)	0.03
Cortical bone thickness (mm)	1.23±0.48 (0.6-2.6)	1.23±0.43 (0.6-2.3)	1.12±0.40 (0.6-2.3)	1.06±0.40 (0.6-2.3)	0.13

**Table 5:** Comparison of HU values at each point. HU values are significantly higher at point A than at points C and D. Steel-Dwass test was used for comparison between each group. p-value<0.05 is considered significant. HU: Hounsfield unit.

HU value	p-value	t-value
Point A vs point B	0.74	1.01
Point A vs point C	0.06	2.51
Point A vs point D	0.04	2.62
Point B vs point C	0.46	1.45
Point B vs point D	0.56	1.31
Point C vs point D	1.00	0.24

**Table 6:** Comparison of HU values between males and females. There is no significant difference. Mann-Whitney U test was used. p-value<0.05 is considered significant. HU: Hounsfield unit.

	Male, mean±SD (range)	Female, mean±SD (range)	p-value
Point A	776.0±216.0 (242.0-1202.2)	775.7±225.9 (538.9-1164.1)	0.96
Point B	714.3±222.9 (297.3-1177.4)	762.2±221.0 (438.5-1288.3)	0.59
Point C	668.9±208.0 (411.6-1164.9)	684.0±185.6 (387.7-1069.4)	0.87
Point D	693.0±211.5 (394.4-1137.1)	671.4±165.7 (488.1-1084.4)	0.87

## Discussion

This study revealed that HU values at the anatomical point were significantly higher than those at the non-anatomical point, and the further proximal from the anatomical point, the smaller the HU values. These results indicate that the anatomical point has better bone quality than the non-anatomical point. In other words, the anchor insertion point proximally away from the anatomical point may lead to anchor dislocation or loosening.

Although good clinical outcomes of arthroscopic ligament repair for CLAI have been reported, recurrent instability is still recognized as an important issue in this procedure, and several factors, such as remnant quality and bony features, were reported [4-8,25]. However, some cases have recurred despite the absence of these factors, and we also consider preventing anchor dislocation or loosening. In one of the problems in the arthroscopic procedure, anchor insertion is frequently enforced in the non-anatomical position, proximal to the anatomical position, because the anatomical position of the fibular attachment cannot be fully observed by arthroscopy [19]. To make it easier to access the anatomical location, we place the accessory portal closer to the anterior margin of the fibula, either slightly distal or closer. Previous reports have demonstrated that anatomical repair yields better surgical results than non-anatomical repair [26,27]. From a biomechanical point of view, Shoji et al. showed that the anatomic ATFL repair state did not show significant differences in kinematics and laxity relative to the intact state; however, the non-anatomic ATFL repair state demonstrated significant inversion, internal rotation kinematics, and internal rotation laxity compared with the intact state [23]. This evidence suggests that an anchor inserted at the non-anatomical point is subjected to abnormal stress, which, together with bone fragility, may lead to anchor dislocation or loosening.

The low HU values of the non-anatomical points in this study may be due to the lack of anatomical structures corresponding to the stress caused by the ligament tension. Suzuki et al. reported that bony trabeculae showed a prominent orientation parallel to the anterior cruciate ligament fiber and that the trabecular orientation was prominent in the proximal-posterior part of the femoral side and the anteromedial part of the tibial side, suggesting that mechanical stress is greater in these parts [28]. FOT is an important indicator for anatomic ligament repair or reconstruction of the lateral ankle ligament [22]. In this study, the 2 mm proximal to the anatomic point of the ATFL had significantly higher HU values than the 8 mm proximal point. In addition, HU values were lower with increasing age at the 4~8 mm proximal points from the anatomical attachment of the ATFL. It is important to insert the anchor within 4 mm of the anatomical attachment of the ATFL to prevent intraoperative or postoperative dislocation or loosening of the anchor. This study used HU values in the CT images to evaluate bone quality quantitatively. Zaidi et al. reported that HU measurement is rapid, simple, and reproducible and that HU values correlate with bone marrow density measurements [21]. Thus, a low HU value area might be considered indicative of low bone mineral density (BMD) and poor bone quality. In shoulder surgery, especially rotator cuff repair, poor bone quality, and osteoporotic bone are known risk factors for the failure of repair, such as suture anchor loosening, impaired tendon healing, and re-rupture of the rotator cuff repair [12-17]. Tingart et al. showed that bone quality significantly impacted anchor failure load, and the proximal part of the greater tuberosity showed higher volumetric BMD and higher pull-out strengths of suture anchors [29]. Thus, a low HU value area might be considered indicative of low BMD and poor bone quality, which leads to anchor dislocation or loosening. As several cases showed lower HU values in this study, even the anatomical attachment, preoperative evaluation of the HU value of the anchor insertion site may be useful to predict anchor problems. 

This study had several limitations. First, the quality of the cortical bone was not evaluated in this study, even though the thickness of the cortical bone was measured. It has been reported that in rotator cuff repair, the failure load of metal anchors reportedly correlates with the BMD of the cortical bone, but not with biodegradable suture anchors. The influence of the cortical bone may be minor when non-metallic anchors are used; however, further investigation is needed. Second, this study involved imaging analysis, and no mechanical tests were performed. Therefore, pull-out strength measurements using biomechanical tests are necessary. Third, there were cases of decreased HU values at the anatomical point, but there was no trend in which cases had decreased HU values. However, preoperative measurement of HU values may be helpful in predicting the risk of anchor dislocation. Further study is needed with more cases. Fourth, the relationship between the HU values, actual anchor position inserted, and recurrence of postoperative instability is unclear. Further studies are required to address these issues. Fifth, regarding the reliability of the HU values, the ICC of the HU value measurement was good in our previous study [30], and the reproducibility of the measurement in this study is considered acceptable. Finally, to avoid unnecessary radiation exposure, there were no postoperative CT scans to confirm the location of the anchors or dislocations.

## Conclusions

This study demonstrated that the anatomical attachment site of the ATFL has higher Hounsfield Unit (HU) values than non-anatomical points, indicating better bone quality. Inserting the anchor away from the anatomical point may increase the risk of dislocation or loosening. The anchor may dislocate or loosen due to weakened bone quality if the anchor is inserted more proximally than 4 mm from the anatomic attachment of the ATFL during arthroscopic repair for CLAI. Preoperative HU assessment could help surgeons identify the optimal insertion site and improve surgical outcomes.
